# Irisin Induces Angiogenesis in Human Umbilical Vein Endothelial Cells *In Vitro* and in Zebrafish Embryos *In Vivo *via Activation of the ERK Signaling Pathway

**DOI:** 10.1371/journal.pone.0134662

**Published:** 2015-08-04

**Authors:** Fei Wu, Haibo Song, Yuan Zhang, Yuzhu Zhang, Qian Mu, Miao Jiang, Fang Wang, Wen Zhang, Liang Li, Huanjie Li, Yunshan Wang, Mingxiang Zhang, Shiwu Li, Lijun Yang, Yan Meng, Dongqi Tang

**Affiliations:** 1 Center for Stem Cell & Regenerative Medicine, The Second Hospital of Shandong University, Jinan, 250012, P.R. China; 2 Jinan Central Hospital Affiliated to Shandong University, Jinan, 250012, P.R. China; 3 Key Laboratory of Cardiovascular Remodeling and Function Research, Qilu Hospital of Shandong University, Jinan, 250012, P.R. China; 4 Department of Pathology, Immunology, and Laboratory Medicine, University of Florida College of Medicine, Gainesville, FL, United States of America; 5 Department of Urology, The Second Hospital of Shandong University, Jinan, 250012, P.R. China; Massachusetts General Hospital/Harvard Medical School, UNITED STATES

## Abstract

As a link between exercise and metabolism, irisin is assumed to be involved in increased total body energy expenditure, reduced body weight, and increased insulin sensitivity. Although our recent evidence supported the contribution of irisin to vascular endothelial cell (ECs) proliferation and apoptosis, further research of irisin involvement in the angiogenesis of ECs was not conclusive. In the current study, it was found that irisin promoted Human Umbilical Vein Endothelial Cell (HUVEC) angiogenesis via increasing migration and tube formation, and attenuated chemically-induced intersegmental vessel (ISV) angiogenic impairment in transgenic TG (fli1: GFP) zebrafish. It was further demonstrated that expression of matrix metalloproteinase (MMP) 2 and 9 were also up-regulated in endothelial cells. We also found that irisin activated extracellular signal–related kinase (ERK) signaling pathways. Inhibition of ERK signaling by using U0126 decreased the pro-migration and pro-angiogenic effect of irisin on HUVEC. Also, U0126 inhibited the elevated expression of MMP-2 and MMP-9 when they were treated with irisin. In summary, these findings provided direct evidence that irisin may play a pivotal role in maintaining endothelium homeostasis by promoting endothelial cell angiogenesis via the ERK signaling pathway.

## Introduction

Angiogenesis, the sprouting of pre-existing vasculature to form new vessels, requires many coordinated endothelial cell activities, such as proliferation, migration, and alignment to form vessel-like tube structures [1–3]. This process, an essential step in tissue repair, occurs in vascular injuries caused by various disorders, such as cardiovascular disease and many other chronic metabolic diseases, especially diabetes [4]. Hence, the factors that can restore injured endothelial cells and stimulate new collateral vessel growth may have important roles in the treatment of vascular damage caused by various diseases. A number of endogenous factors and hormones have been reported to participate in the regulation of angiogenesis [5].

Exercise is the frontline defense for prevention of many cardiovascular and metabolic diseases. Irisin, secreted by skeletal muscles in response to PGC-1α during exercise, is a cleaved and secreted fragment of fibronectin type III domain containing protein 5 (Fndc5), a type I transmembrane protein of skeletal muscles which can be up-regulated by PGC-1α and exercise [6]. As a link between exercise and metabolism, irisin is thought to be involved in increased total body energy expenditure, reduced body weight, and increased insulin sensitivity in mice [6, 7]. A previous study from our laboratory revealed its mechanism [8]. Several reports have shown that circulating irisin levels are significantly lower in type 2 diabetes mellitus (T2DM) patients, which means that serum irisin might be a new marker of T2DM [9–11]. In addition to studies of irisin in adipose tissue and metabolic homeostasis, the circulating irisin level might have a role in other systems, such as cardiovascular and central nervous systems [12, 13]. Interestingly, a recent study discovered that pharmacological concentrations of irisin increased proliferation of H19-7 hippocampal neuronal cells [13]. Another study demonstrated the potential role of irisin in bone metabolism via modulating osteoblast differentiation [14]. These discoveries also suggest that the physiology of irisin in humans is still far from completely understood. In our previous study, we demonstrated the effect of irisin in promoting HUVEC proliferation via the ERK signaling pathway and that it partially protects the cell from high glucose-induced apoptosis [15]. These finding suggest that there is a link between irisn and the vascular endothelium. However, no previous studies have evaluated whether irisin directly regulates other functions of human ECs such as migration and angiogenesis.

In the present study, HUVEC and transgenic TG (fli1: GFP) zebrafish were treated with human recombinant irisin (r-irisin) which was expressed and purified in our laboratory [8]. HUVEC migration and cord formation, as well as ISV formation of zebrafish, were evaluated for the effects of irisin. Also, irisin treatment led to MMP-2 and MMP-9 up-regulation in endothelial cells. The signaling pathways involved in this process were also characterized. For the first time, these studies demonstrated that irisin exerts its influence in endothelial cell angiogenesis via the ERK pathway. This opens crucial avenues that could be applied to the regeneration process and cure of vascular disorders.

## Materials and Methods

### Expression and Purification of Human Irisin

The production of human irisin was performed as previously described [8]. Briefly, human irisin cDNA (360bp) was synthesized by Life Technologies. After cloning the cDNA into EcoR1/Xba1 sites of the pPICZαA plasmid, the linearized pPICZαA-irisin was transformed into *Pichia pastoris* X-33. The resulting transformant was cultured and induced as previously described [16]. The recombinant irisin was first precipitated via addition of ammonium sulfate and then further purified by loading the precipitated protein onto a concanavalin A–agarose column as introduced by our previous study [8].

### Cell Culture of Human Umbilical Vein Endothelial Cells (HUVEC)

Human umbilical vein endothelial cells (HUVEC) were isolated from freshly obtained human umbilical cords as previously described [15]. The research was carried out in strict accordance with the ethical guidelines of the 1975 Declaration of Helsinki. Approval for this study was obtained from the Institutional Medical Ethics Committee of Qilu Hospital, Shandong University. All donors provided written informed consent in this study. The HUVEC were cultured in Medium 199 (M199, Invitrogen) supplemented with 10% FBS (Invitrogen), 10 ng/mL EGF, and 10 ng/mL bFGF (Peprotech) at 37°C in a humidified atmosphere with 5% CO_2_.

### Cell Viability and Proliferation Assay

Cell viability was assessed by using the MTT Cell proliferation and cytotoxicity assay kit (Beyotime, China). Briefly, HUVEC were seeded into 96-well plates (100 μl/well of 3 × 10^4^ cells/ml). After incubation with 20 nM irisin, 5 ug/ml mitomycin C, or 20 nM irisin with 5 ug/ml mitomycin C for different time-points (0, 6, 12, 24, and 48 h), 10μl MTT (5 mg/ml) was added into each well and incubated at 37°C for 4 h. Then 100 μl of formanzan lysis was added for another 4 h. Finally, the absorbance was measured with a microplate reader (Bio-tek) at a wavelength of 570 nm.

### *In Vitro* Wound-Healing Migration Assay

The wound-healing assay was performed as previously reported [17]. HUVECs were seeded in 6-well plates at 1×10^6^ / well in M199 containing 10% FBS, 10 ng/ml bFGF, and 10 ng/ml EGF. After growth to 80%- 90% confluence, a horizontal line was created by scraping cells with a sterile disposable pipette tip to make a scratch wound. Then the cells were washed twice with phosphate buffered saline (PBS) and incubated in M199 with 2% FBS, 5 μg/ml mitomycin C and different concentrations of irisin. After incubation for 12 h and 24 h, the cells that had migrated to wound areas were photographed with an inverted phase contrast microscope (AMG EVOS fl, Bothell, WA).

### Transwell Chamber Migration Assay

The capacity of HUVEC to migrate was determined using a Transwell Boyden chamber (Corning Costar, Cambridge, MA, USA) with 8.0 μm pore-size polycarbonate filters inserts in 24-well plates as previously described [18, 19]. Cells (5× 10^6^/ mL, 100 μL) were seeded on the upper compartment of the chamber in the presence or absence of different concentrations of irisin with 5 μg/ml mitomycin C in serum-free M199. The lower compartment contained 600 μL of M199 supplemented with 10% FBS to stimulate cell migration. After incubation for 24 h at 37°C, the filter was removed and fixed by formaldehyde (3.7% in PBS) at room temperature for 20 min and then stained by 1% crystal violet at room temperature for 15 min, and the non-migrating cells were scraped off the upper chamber with cotton swabs. The membranes were washed twice in PBS and cells on the lower surface were counted and photographed with a fluorescent microscope (Olympus; Tokyo, Japan) in 5 random fields.

### Matrigel Tube Formation Assay

The angiogenic capacity of HUVEC on Matrigel was determined according to the manufacturer’s recommendation. After being incubated on ice, the BD Matrigel matrix was plated in a pre-cooled 12-well chamber. After solidification of Matrigel at 37°C, 5% CO_2_ for 1 h, HUVEC were trypsinized and seeded (5×10^4^ cells per well) in each well with 5 μg/ml mitomycin C (control sample) or 5 μg/ml mitomycin C with irisin in different concentrations. Each sample was added to duplicate wells. The chambers were then incubated at 37°C for 6 h. Tube formation was observed using an inverted phase contrast microscope (AMG EVOS fl, Bothell, WA).

### Zebrafish Angiogenesis Assay

The zebrafish line TG (fli1: GFP) with transgenic endothelial cells expressing GFP was obtained from the Biology Institute of Shandong Academy of Sciences. The animals were maintained in a constant aeration and flow water systems under a 14 h light/10 h dark photoperiod. Zebrafish maintenance and experimental operations were conducted in accordance with National Institutes of Health guidelines of the use and care of experimental animals and approved by the Institutional Animal Care and Use Committee of Shandong University.

Zebrafish embryos were collected from natural pair-wise matings. At 24 h post-fertilization (hpf) embryos were manually dechorionated and incubated with 6 μM of PTK787 (Sigma) in 24-well plates (8–10 embryos/well). After treatment with PTK787 for 4 h, embryos were washed three times and exposed in water to 20, 50 and 100 nM of irisin together with PTK787 (6 μM) for another 20 h, while embryos receiving only 0.4%v/v DMSO or PTK787 (dissolved in 0.4%v/v DMSO) for 24 h acted as positive and negative controls. To analyze the pro-angiogenesis effect of irisin, the morphology of the intersegmental vessel (ISV) region was visually assessed by using an Olympus SZX16 florescent microscope after 48 hpf. For quantification comparisons, the total length of the ISVs per larva was measured for ten ISVs located anterior and posterior of the yolk sac extension. While the zebrafish were photographed under the microscope, 0.05% 2-phenoxyethanol anesthesia was implemented to ameliorate suffering.

### RNA Extraction and Quantitative Reverse Transcription-PCR

Total RNA was isolated from the cells and zebrafish embryos with TRIzol reagent (Invitrogen, Carlsbad, CA), and then reverse transcribed to cDNA with a SuperScript II reverse transcription kit (Invitrogen, Carlsbad, CA) according to the manufacturer's instructions. PCR amplification was run using a PCR machine (Bio-Rad, Hercules, CA, USA). A standard amplification protocol was used for real-time PCR analysis: 95°C for 10 s, followed by 40 cycles of 95°C for 5 s, 60°C for 30 s, and a final extension at 72°C for 3 min. Primers that were used for qPCR are summarized in the Table 1. After amplification, CT values were extracted and ΔΔCt values were calculated to determine the relative abundances of amplification. Experiments were performed in triplicate.

**Table 1:** Primers for qPCR.

Primers	Forward; 5’—3’	Reverse: 5’—3’	Product length (bp)
**Human MMP-2**	GATACCCCTTTGACGGTAAGGA	CCTTCTCCCAAGGTCCATAGC	**112**
**Human MMP-7**	GGA TGG TAG CAG TCT AGG GAT TAA CT	GGA ATG TCC CAT ACC CAA AGA A	**79**
**Human MMP-9**	AGACCTGGGCAGATTCCAAAC	CGGCAAGTCTTCCGAGTAGT	**94**
**Human MMP-13**	TCCTGATGTGGGTGAATACAATG	GCCATCGTGAAGTCTGGTAAAAT	**184**
**Human TIMP1**	ACCACCTTATACCAGCGTTATGA	GGTGTAGACGAACCGGATGTC	**96**
**Human endostatin**	TGGTCTACGTGTCGGAGCA	GCCTCGTTCGCCCTTAGAG	**128**
**Human β-actin**	CATGTACGTTGCTATCCAGGC	CTCCTTAATGTCACGCACGAT	**250**
**Zebrafish MMP-2**	GGACATCTCGGAATCGGACC	GCAGGGAAGCAAGAAACTGC	**99**
**Zebrafish MMP-9**	TTGGCTTCTGTCCCAGTGAG	TTAGGGCAGAATCCATACTT	**191**
**Zebrafish β-actin**	ACTCAGGATGCGGAAACTGG	AGGGCAAAGTGGTAAACGCT	**118**

### Western Blot Analysis

HUVEC were seeded into 6-well plates at 1×10^6^/well in M199 containing 10% FBS, 10 ng/ml bFGF, and 10 ng/ml EGF. After starvation in serum-free media for 24 h, the cells were treated with or without irisin. HUVEC were washed twice with ice-cold PBS and then lysed in RIPA lysis buffer supplemented with a protease inhibitor cocktail. The total cell protein concentration was determined using the BCA protein assay kit (Pierce, Rockford, IL, USA) according to manufacturer’s instructions. Equal amounts of total protein were loaded into and separated by 12% sodium dodecyl sulfate polyacrylamide gels (SDS-PAGE) and subsequently transferred to polyvinylidene fluoride (PVDF) membranes. After blocking with 5% fat-free milk for 1 h at room temperature, the membranes were incubated with 1:1000 dilution primary antibodies overnight at 4°C. Anti-ERK1/2 (#4695), anti-phospho-ERK1/2 (#9101), anti-Akt (#9272), anti-phospho-Akt (#4058), anti-P38 MAPK (#9212), and anti-phospho-P38 MAPK (#4631) were purchased from Cell Signaling Technology, and anti-MMP2 (sc-13594), anti-MMP9 (sc-21733) and anti-β-actin (sc-7210) were purchased from Santa Cruz Biotechnology. Then, the membranes were washed three times with TBS and 0.1% Tween-20, and incubated with goat anti-rabbit IgG-HRP (1:10000; sc-2004, Santa Cruz) or goat anti-mouse IgG-HRP (1:10000; sc-2005, Santa Cruz) for 1 h at room temperature. Finally, the membranes were developed in an enhanced chemiluminescence system with an ECL kit, and the relative intensity was quantified by densitometric analysis using the Alpha Imager 2200.

### Gelatin Zymography

MMP-2/MMP-9 protein secretion and activation in conditioned medium were visualized by electrophoresis on gelatin-containing polyacrylamide gel, as previously described[20]. HUVECs were seeded in 6-well plates and cultured in 5% CO_2_ at 37°C until 80% confluence. Subsequently, the medium was replaced by 2 mL of serum-free M199 alone (control sample) or M199 supplemented with irisin in different concentrations for 24 h. After 24 h of incubation, the conditioned supernatants were collected and used for electrophoresis on a 10% SDS-PAGE containing 0.1% (w/v) gelatin A (Sigma-Aldrich, St. Louis, MO, USA). After electrophoresis, the gel was washed 3 times for 30 min in zymography renaturing buffer (2.5% Triton X-100), and then incubated for 18 h at 37°C in a buffer solution containing 50 mmol/L Tris-HCl (pH 7.4), 200 mmol/L NaCl, and 5 mmol/L CaCl_2_. The gel was then stained with Coomassie Brilliant Blue (0.1% Coomassie Brilliant Blue R-250, 30% methanol, and 10% acetic acid in H_2_O) and destained with 30% methanol, 10% acetic acid in H_2_O until clear bands suggestive of gelatin digestion were present.

### Statistical Analysis

The data are presented as the mean ± standard deviation (SD). Each experiment was repeated at least of 3 times. Student’s t test was used for comparing differences of different groups. Comparisons among values of multiple groups were performed by one-way analysis of variance (ANOVA). Statistical significance was set at P<0.05.

## Results

### Irisin Promotes Migration in HUVEC

**Fig 1:**
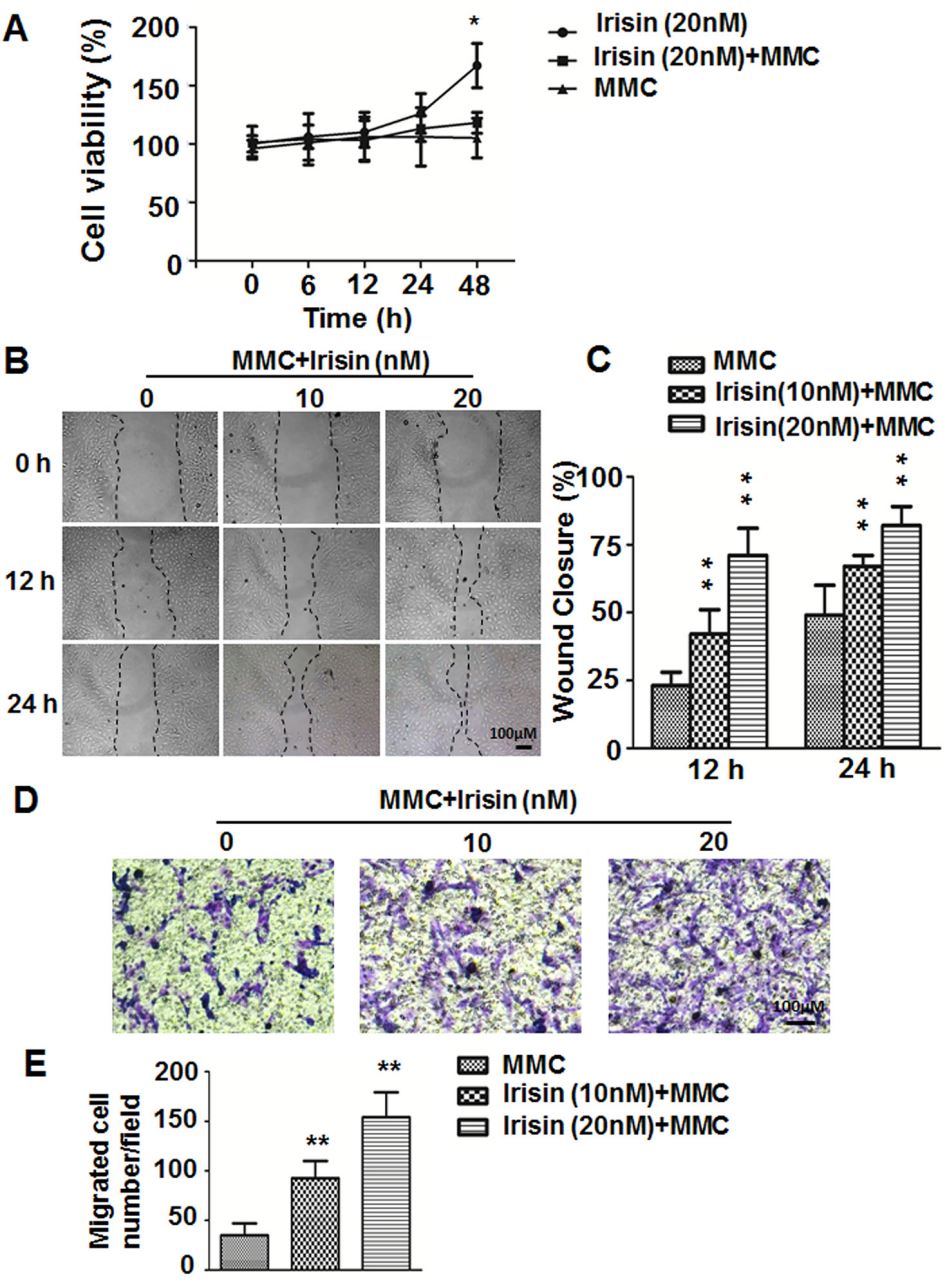
Effect of irisin on migration of HUVEC. (A) After HUVEC were incubated with 20 nM irisin, 5 μg/ml mitomycin C (MMC), or 20 nM irisin with 5 ug/ml MMC for different time periods (0, 6, 12, 24, and 48 h), MTT assay was used to detect the viability of the cells. * P< 0.05 vs. irisin (20 nM) +MMC group, determined by unpaired two-tailed Student’s t-test. (B) Images of wound-healing assays of HUVEC at 0, 12 and 24 h after scratch wound. (C) Percent wound closure at 12 and 24 h after scratch is shown (n = 5). (D) Images of Transwell assays of HUVEC treated with or without irisin (10 and 20 nM). (E) The number of HUVEC that migrated to the lower side of the membranes after incubation for 24 h are shown and the relative migration was quantified using Image-Pro Plus software (n = 5). Data are mean± SE. ** P< 0.01 vs. MMC treated group, determined by unpaired two-tailed Student’s t-test.

According to our previous study [15], irisin can promote HUVEC proliferation and the maximum effect of irisin on HUVEC proliferation appeared when its concentration was 20 nM. To exclude the influence of irisin-induced proliferation, mitomycin C was used. As shown in Fig 1A, irisin-induced cell proliferation was significantly inhibited in the mitomycin C co-administration group. So we added mitomycin C for the subsequent migration and tube formation experiments.

A wound healing assay was used to assess the migration of HUVEC. Compared with the control group, the wound healing of HUVEC was gradually accelerated in the groups treated with 10 nM and 20 nM irisin at both 12 h and 24 h (Fig 1B and 1C). The Transwell chamber migration assay revealed that after treatment with irisin at concentrations of 10 nM and 20 nM for 24 h, HUVEC showed a significant increase in migratory ability. The migrating cell numbers were 35±12.1 (control), 92.7±17.2 (10 nM), and 154±25.2 (20 nM) per high power field (Fig 1D and 1E), and the difference was statistically significant (P < 0.01).These results indicated that irisin has the ability to enhance endothelial cell migration.

### Irisin Promotes Angiogenesis in HUVEC

**Fig 2:**
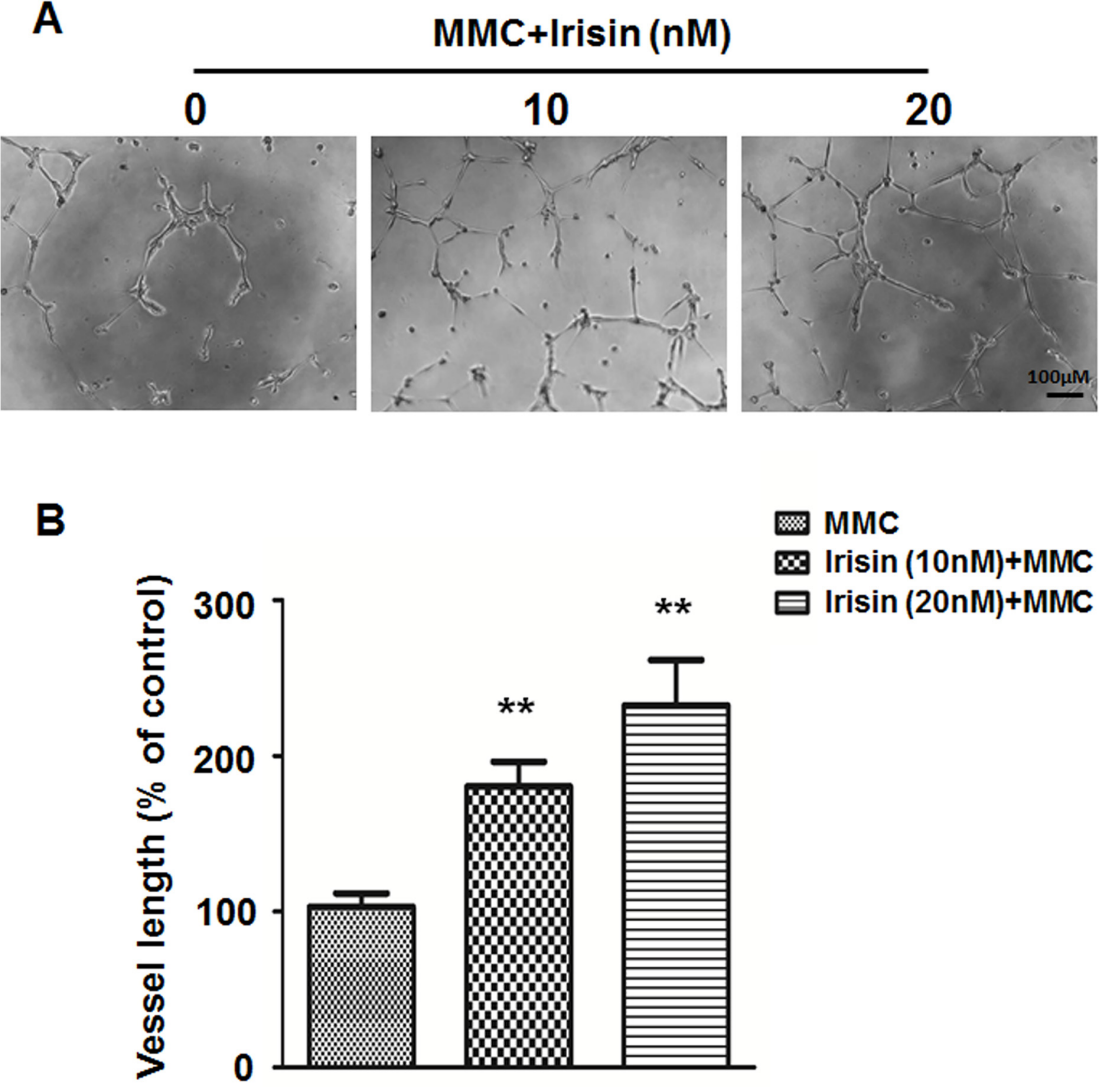
Irisin stimulates angiogenesis of HUVEC. (A) Effect of irisin (10 and 20 nM) on angiogenesis of HUVEC was measured by *in vitro* tube formation assay. (B) Tube formation after 6 h incubation on Matrigel was quantified by measuring the vessel length in randomly selected fields (n = 5). Data are presented as the mean± SE. ** P< 0.01 vs. MMC treated group, determined by unpaired two-tailed Student’s t-test.

To investigate the effect of irisin on angiogenic tubule formation, HUVEC were seeded on a Matrigel substrate with either 5 μg/ml mitomycin C or mitomycin C supplemented with irisin in concentrations of 10 nM and 20 nM. The results showed that the presence of irisin induced a significant increase in capillary-like tube formation after just 6 h compared with the control (Fig 2A and 2B).

### Irisin Attenuated Chemically-Induced Blood Vessel Damage in Zebrafish

**Fig 3:**
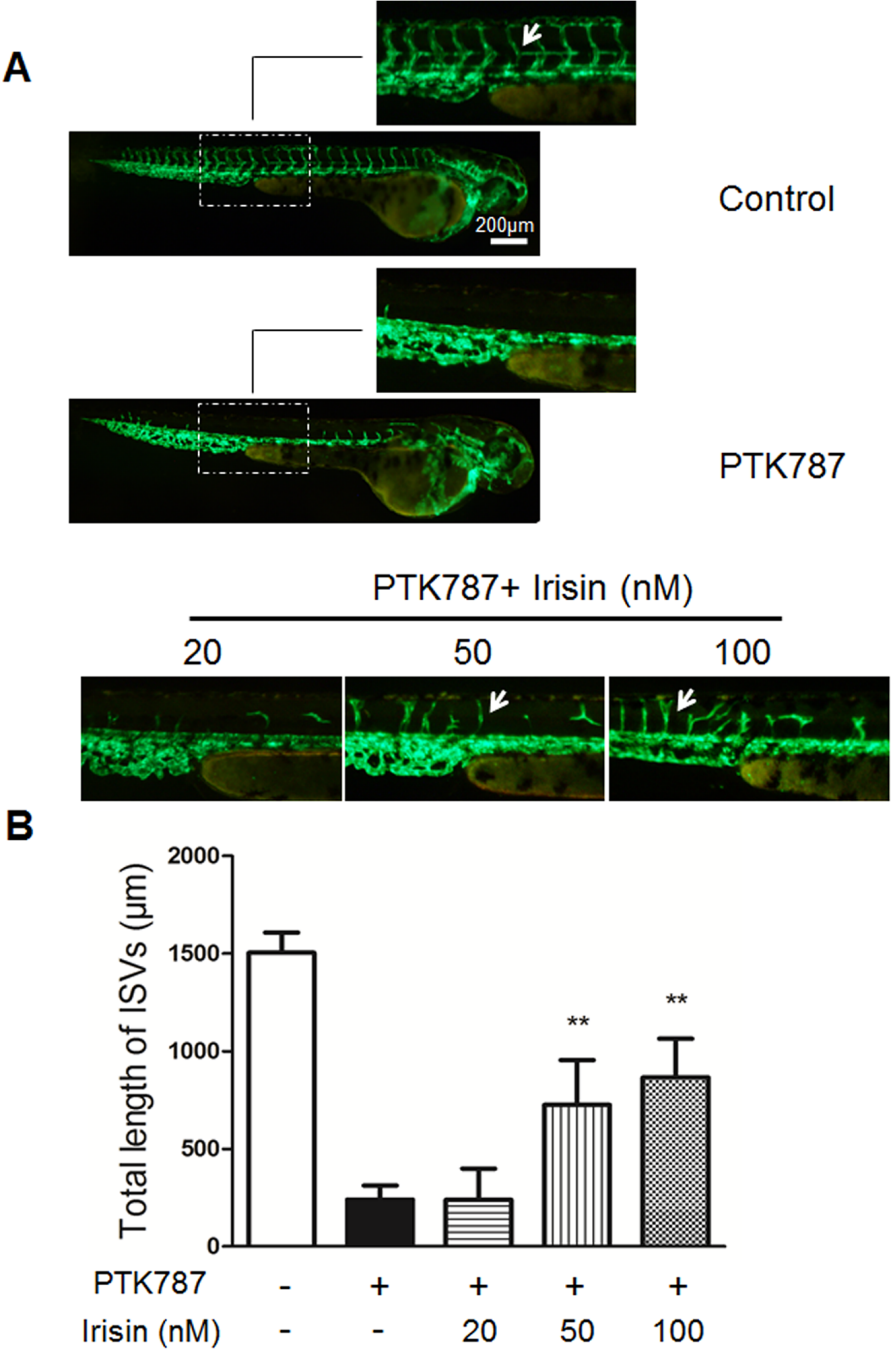
Irisin restored chemically-induced ISV angiogenesis impairment in zebrafish. (A) Lateral view of control embryos and embryos treated with 6 uM of PTK787 for 24 h. Embryos treated with 20, 50, and 100 nM of irisin after pretreatment with PTK787. ISVs are indicated by the white arrows. (B) Quantification of total length of ISVs induced by irisin (n = 6–8). Data are presented as the mean± SE. **P< 0.01 vs. PTK787 treated group, determined by unpaired two-tailed Student’s t-test.

The *in vivo* angiogenic effect of irisin was studied by measuring the length of the intersegmental vessel (ISV) of transgenic zebrafish by fluorescent microscopy. In zebrafish without blood vessel damage, the ISV showed no significant changes after treatment with irisin for 24 h compared with the vehicle group (Data not shown). So we used PTK787 to produce a chemically-induced blood vessel damage model to investigate the pro-angiogenic effect of irisin under this circumstance. As a potent inhibitor of vascular endothelial growth factor (VEGF) receptor tyrosine kinases, PTK787 displays an anti-angiogenic property in a dose-dependent manner [21]. Also, exposure to PTK787 can lead to concentration-dependent reductions in ISVs length of zebrafish embryos [22]. Fig 3 shows the total length of ten ISVs for each larva in different groups at 48 hpf. The ISVs length were 1504.18±103.30 μm in the group of control and 240.14±71.31 μm in the PTK787 treated group, which indicated that PTK787 alone significantly blocked the formation of ISVs. In the PTK787-pretreatment plus irisin groups, the length of ISVs were 239.91±159.01 μm, 725.37±229.31 μm and 865.39±199.60 μm following 20, 50 and 100 nM irisin treatment (Fig 3A and 3B). Taken together, these results suggested that irisin in the dose of 50 and 100 nM could attenuate chemically-induced blockage of ISV formation in zebrafish.

### Irisin Up-Regulates Expression and Activity of MMP-2 and MMP-9

**Fig 4:**
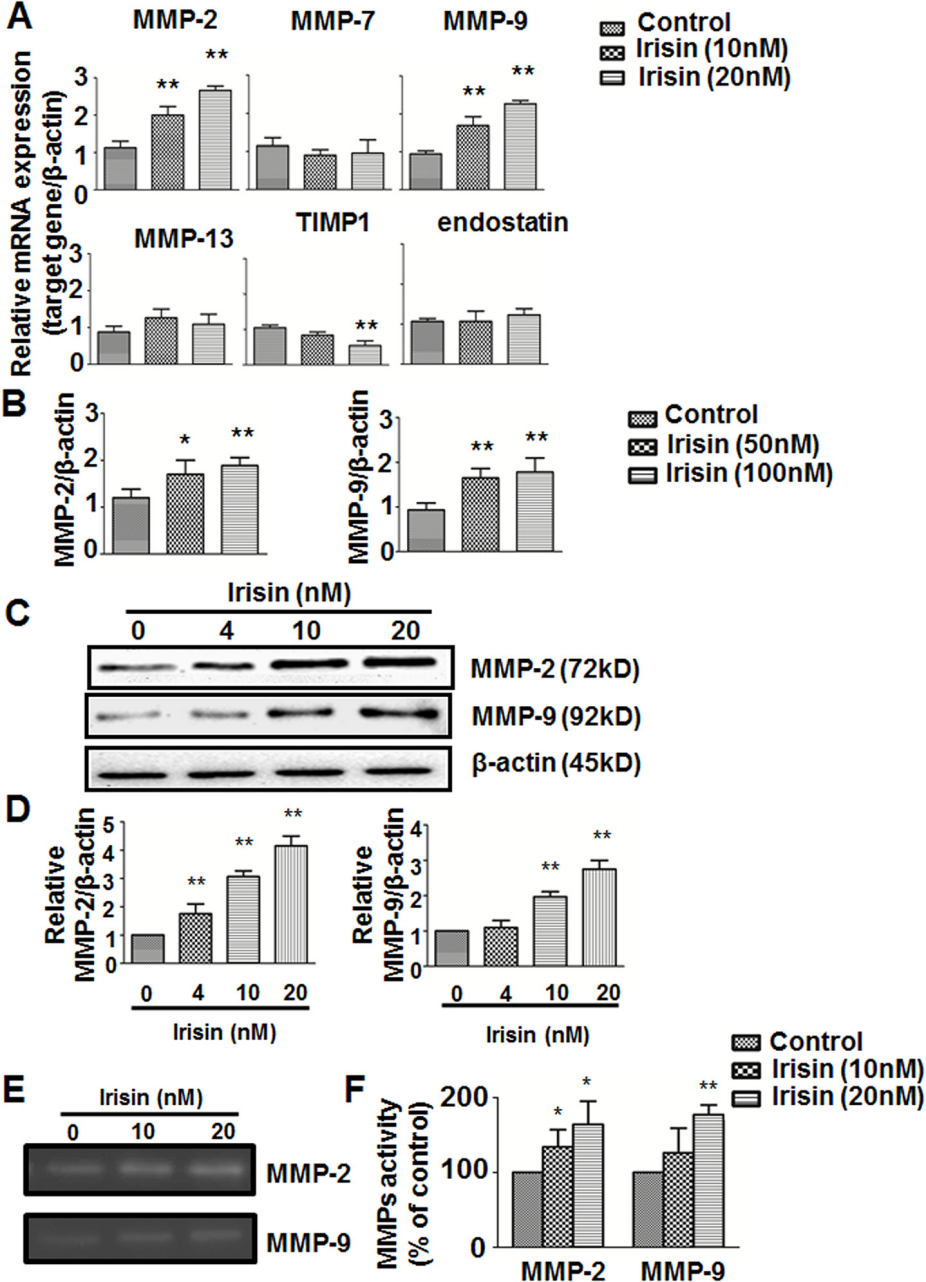
Effect of irisin on matrix metalloproteinases (MMPs) expression and gelatinolytic activity (A) qPCR analysis of MMPs, TIMP1, and endostatin mRNA expression after treatment with or without irisin (10 and 20 nM) for 24 h (n = 3). (B) qPCR analysis of MMPs expression in zebrafish embryos after treatment as described in methods and materials (n = 3). (C) Expression of MMP-2 and MMP-9 were determined by Western blot and normalized with the β-actin level. (D) and (F) Protein bands were quantified by densitometric analysis. (E) MMP-2 and MMP-9 activities were analyzed by gelatin zymography. Data are presented as the mean± SE of triplicates. ** P< 0.01 vs. control group, determined by unpaired two-tailed Student’s t-test.

Mounting evidence supports the view that matrix metalloproteinases (MMPs) mediate the process of remodeling the extracellular matrix (ECM) and is a prerequisite for angiogenesis [19, 23]. To determine which MMPs can be influenced by irisin in HUVEC, we examined mRNA expression of MMPs and other genes which may play a critical role in the progress of cells migration. The quantitative real-time PCR results showed that irisin significantly stimulated expression of MMP-2 and MMP-9 and slightly reduced expression of TIMP-1 in HUVEC (Fig 4A). TIMPs play a role in regulating MMP activation in several different tissues and suppress the activity of MMPs [24]. The q-PCR results of MMPs expression in zebrafish embryos were also in line with what we found in HUVEC (Fig 3B). To verify the real-time PCR results, Western blot showed that MMP-2 and MMP-9 protein levels were also highly up-regulated after treatment with irisin for 24 h (Fig 4C and 4D).Gelatin-zymography analysis showed that irisin potently increased the gelatinolytic activity of MMP-2 and MMP-9 secreted from HUVEC (Fig 4E and 4F).

### Irisin Mediates HUVEC Migration and Tube Formation through the ERK Signaling Pathway

**Fig 5:**
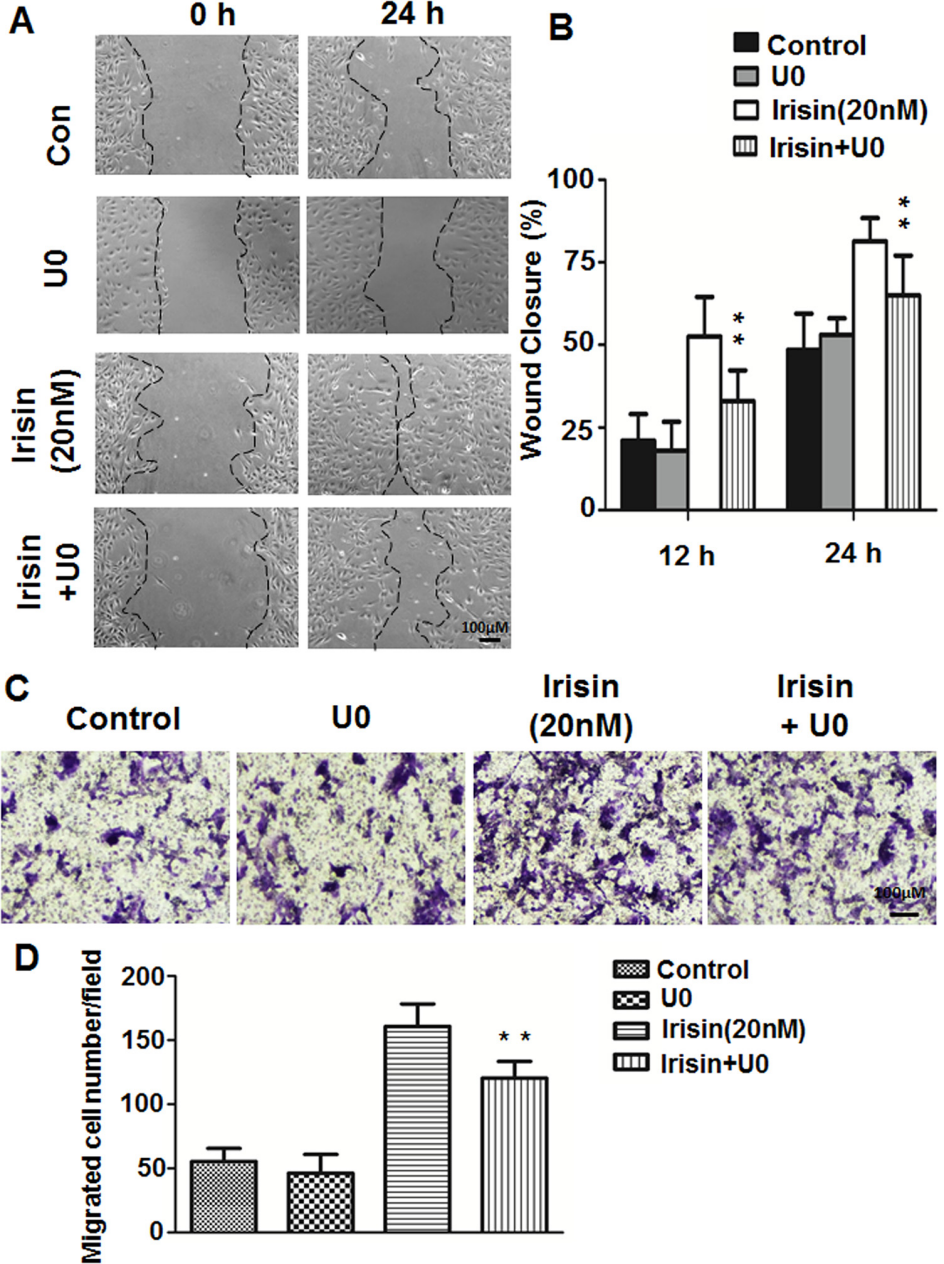
Effect of the ERK inhibitor on irisin-induced cell migration of HUVEC. (A) and (C) HUVEC were pretreated with the ERK inhibitor U0126 (10 μM) for 30 min and cultured with or without irisin (20 nM) for 24 h, images of wound-healing assays and Transwell assays were taken at 0 and 24 h. (B) Percent wound closure at 12 and 24 h after scratch is shown (n = 5). (D) The number of cells that migrated to the lower side of the membranes after incubation for 24 h are shown and relative migration was quantified using Image-Pro Plus software (n = 5). Data are mean± SE. ** P< 0.01 vs. irisin-treated group, determined by unpaired two-tailed Student’s t-test.

**Fig 6:**
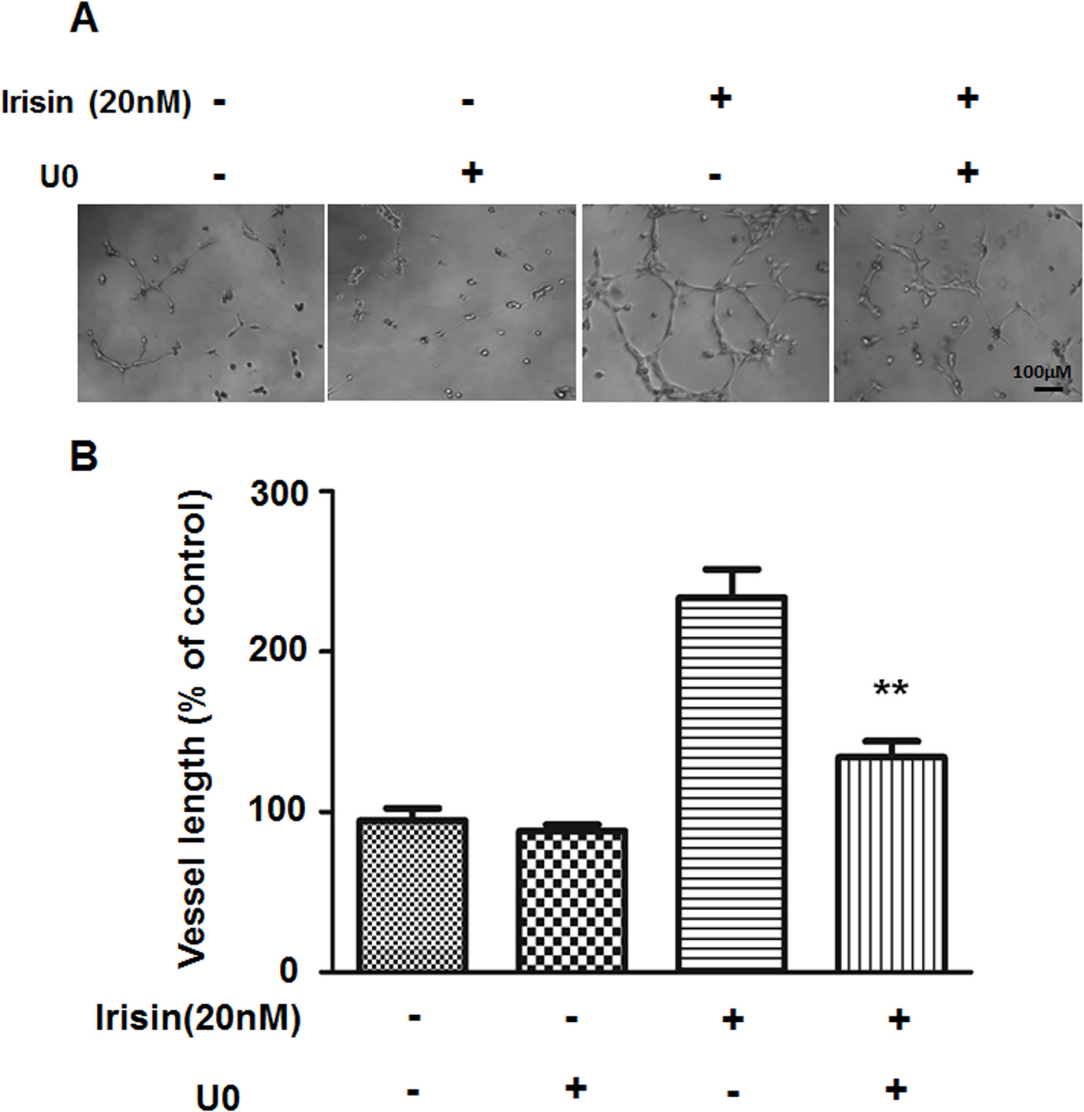
Effect of the ERK inhibitor on irisin-induced angiogenesis of HUVEC. (A) HUVEC were pretreated with the ERK inhibitor U0126 (10 μM) for 30 min and cultured with or without irisin (20nM), images of Matrigel tube formation assay were taken after incubation for 6 h. (B) Tube formation was quantified by measuring the vessel length in randomly selected fields (n = 5). Data are presented as the mean± SE. **P< 0.01 vs. irisin-treated group, determined by unpaired two-tailed Student’s t-test.

We demonstrated that ERK, not p38 MAPK or AKT signaling pathways were involved in irisin-induced HUVEC proliferation and apoptosis [15]. It was confirmed that phosphorylated ERK (P-ERK) was significantly increased after treatment with irisin for 5 min and 10 min (S1A Fig). To explore whether ERK is involved in HUVEC migration and tube formation, a specific inhibitor of the ERK pathway, U0126, was used to block ERK activation. As shown in Figs 5 and 6, irisin-induced cell migration and tube formation was significantly inhibited by 10 μM U0126. Also, Western blot showed that the irisin-induced increase in ERK phosphorylation was inhibited by pre-treatment with U0126 (S1B and S1C Fig).

### Effects of the ERK Inhibitor on Irisin-Induced Expression and Activity of MMP-2 and MMP-9

**Fig 7:**
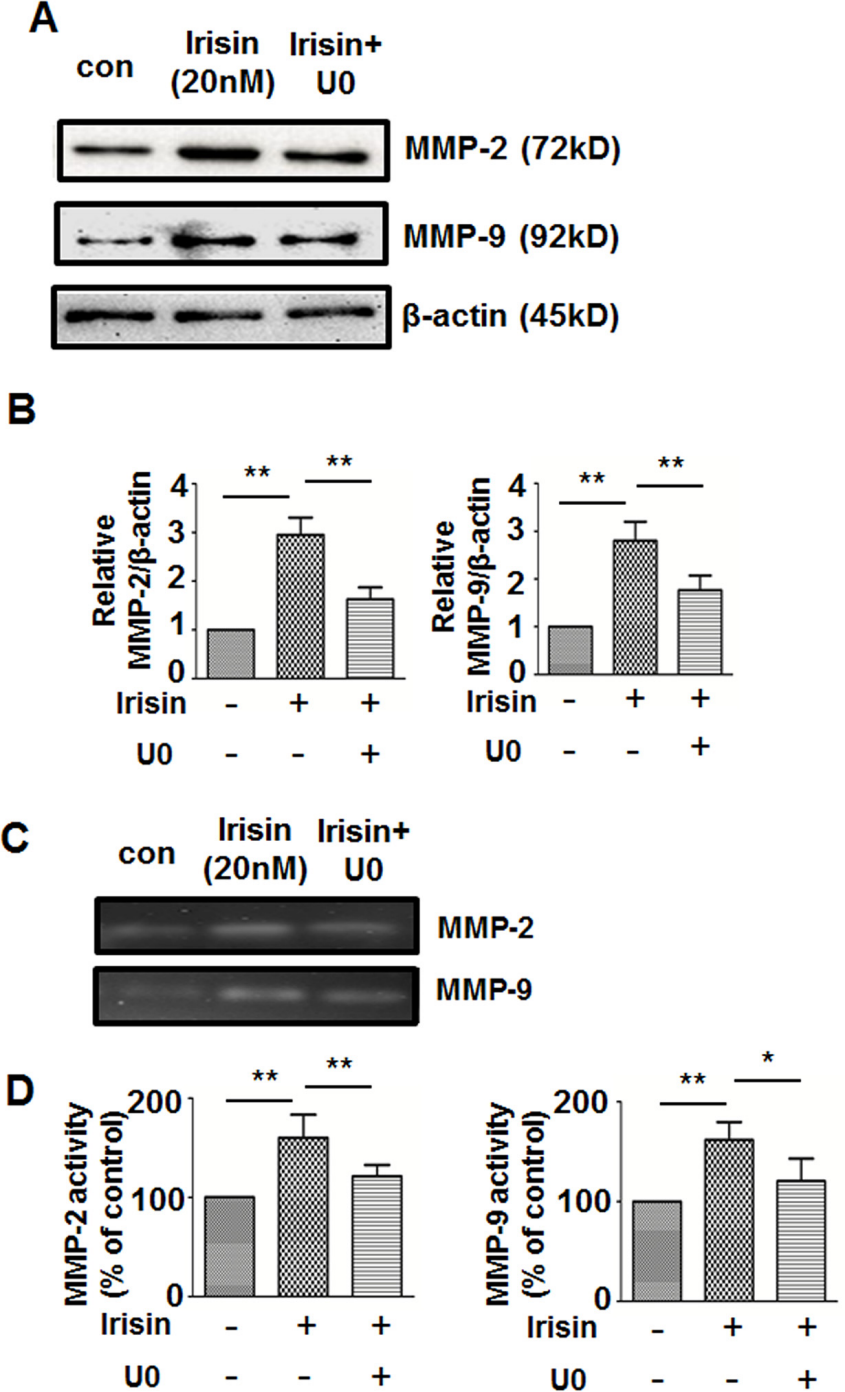
Effect of U0126 on the irisin-induced expression and gelatinolytic activity of MMP2/9 in HUVEC. HUVEC were pretreated with the ERK inhibitor U0126 for 30 min followed by irisin treatment (20 nM) for an additional 24 h, then the MMP-2, MMP-9 and β-actin protein expression were analyzed by Western blot (A) and MMP-2/9 activities were analyzed by gelatin zymography (C). (B) and (D) Protein bands were quantitated by densitometric analysis. Data are presented as the mean± SE of triplicates. ** P< 0.01,* P< 0.05, determined by unpaired two-tailed Student’s t-test.

S1A Fig shows that irisin treatment induced ERK phosphorylation. We sought to identify whether ERK participates in irisin-induced MMP-2 and MMP-9 up-regulation. After treatment with U0126 for 24 h, the irisin-induced increase in protein expression and gelatinolytic activity of MMP-2 and MMP-9 was significantly inhibited (Fig 7). These results suggested that ERK is pivotal in irisin-induced up-regulation of MMP-2 and MMP-9.

## Discussion

The first recorded report of angiogenesis was in 1794 by the Scottish anatomist and surgeon John Hunter [25]. In the past 40 years the field of angiogenesis has grown enormously because stimulation of angiogenesis can be therapeutic in several life-threatening medical conditions. Reduction or interruption of angiogenesis is closely associated with the pathological process of several cardiovascular diseases, peripheral arterial disease, and chronic metabolic diseases. Ischemic heart disease (IHD) often occurs because of the interruption of blood flow due to acute occlusion of major coronary arteries [26]. Hence, potential therapeutic strategies for IHD focus on enhancing angiogenesis and neovascularization in order to restore blood flow to the myocardium surrounding the infarct area. As one of the major deteriorating factors of coronary and peripheral artery diseases, the development of diabetes has been widely recognized as having a close relationship to both macrovascular and microvascular complications [27]. Acute hyperglycemia also impairs endothelium-dependent vasodilation and triggers endothelial dysfunction by decreasing production of its trophic factors [28]. Hence, therapeutic strategies designed to increase the neovascularization process and augment native collateral vessel blood flow may be of value for the treatment of diabetes-induced vascular damage.

In 2012, the study by Bostrom et al. showed that irisin, a segment of FNDC5 released from skeletal muscle following exercise, is implicated as a link between exercise and metabolic homeostasis. They reported that irisin enhances the “browning” of white adipocytes in mice, and this process increases total body energy expenditure, reduces body weight, and increases insulin sensitivity [6, 7]. In addition to its potent metabolic effects, more and more findings have revealed that irisin may have a role in other systems. It has been reported that increased serum irisin levels in patients with established coronary artery disease after percutaneous coronary interventions are associated with future major adverse cardiovascular events [12]. In the central nervous system, FNDC5 knockdown significantly decreased the neural differentiation rate of mouse embryonic stem cells [29]. Gannon et al. found that irisin may offer therapeutic benefits for breast cancer prevention by decreasing malignant cell viability and synergizing with doxorubicin [30]. Moreover, irisin is associated with bone density and strength in athletes and can enhance the differentiation of bone marrow stromal cells into mature osteoblasts [14, 31]. All of these data indicate that irisin, this relatively little known myokine, needs to be deeply studied to clarify its full potential effects as a valuable drug target in human disease states.

Previous study of our laboratory demonstrated the pro-proliferation and anti-apoptotic effects of irisin on HUVEC [15]. However, irisin has never been indicated as a pro-angiogenic factor of the vascular endothelium. In the current study, the positive impact of irisin on angiogenesis both *in vitro* and *in vivo* was investigated. The results showed that application of irisin potently promotes angiogenesis via increasing migration and formation of capillary-like structures in HUVEC by up-regulating protein and mRNA expression and gelatinolytic activity of MMP-2 and MMP-9. Also, irisin protected against chemically-induced angiogenesis impairment in zebrafish. It was also found that irisin activated the ERK signaling pathway in this process. However, ERK activation was blocked by using its inhibitor U0126 which mitigated irisin-mediated migration and tube formation in HUVEC.

Many angiogenic factors and hormones have been reported to participate in the regulation of angiogenesis, such as vascular endothelial growth factor (VEGF) and matrix metalloproteinases (MMPs). MMPs are crucial for endothelial cells in the degradation of the basement membrane in order for cells to migrate into surrounding tissues. Among these, the role of MMP-2 and MMP-9 have been emphasized because their type IV collagenase activity is essential during the initial phase of angiogenesis [24]. Therefore, our findings that irisin-induced up-regulation of MMP-2 and MMP-9 may show a potential causal relationship between irisin induced angiogenesis and MMPs activity.

Several studies have reported that certain signaling pathways can lead to expression of a particular MMP gene and potentially the promotion of angiogenesis [24]. Fucoidan/FGF-2 induced angiogenesis and expression of MMP-2 in HUVEC is mediated by p38 and JNK [32]. Both fibronectin and vitronectin induce MMP-9 expression via AP-1-activating signaling pathways through integrin α5β1/αvβ3 dependent Akt, ERK, and JNK signaling pathways in endothelial cells [33]. As one subset of the classical mitogen-activated protein kinases (MAPKs), ERK signaling is critical for growth and survival of cells by regulating the processes of proliferation, differentiation, and migration in a variety of cells [34, 35]. Our results, as well as the previous reports from our laboratory [8, 15], showed that irisin up-regulated expression of the ERK signaling pathway. Moreover, the data also showed that pretreatment with the ERK inhibitor U0126 significantly suppressed irisin-induced cell migration and tube formation, as well as the increased expression and activity of MMP-2 and MMP-9. These data indicated for the first time the role of the ERK signaling pathway in irisin-induced MMPs up-regulation, as well as endothelial migration and tube formation. However, although our results indicated that the increased ERK phosphorylation was almost completely abolished by the ERK inhibitor U0126, it still cannot completely block the effects of irisin on cells migration and tube formation. This suggested that other pathways may also be involved in this process. For instance, Park et al. reported that integrin αvβ3 and AMPK mediate recombinant human CCN1-induced endothelial cell angiogenesis [36]. PP1cβ is considered to be critical in regulating endothelial cell migration and the angiogenic process [37]. Other molecules, such as NF-κB and PPARs have also been suggested as regulators in promoting angiogenesis [38–40]. Thus, we suggest that the ERK signaling pathway plays a crucial role in irisin-induced endothelial cell migration and angiogenesis, but further studies on the underlying mechanisms should still be considered.

In conclusion, the current study indicated that irisin promoted angiogenesis in HUVEC *in vitro* and its pro-angiogenic effect was also confirmed *in vivo* by attenuating chemically-induced ISV angiogenic impairment in zebrafish. Moreover, irisin can up-regulate mRNA and protein expression and gelatinolytic activity of MMP-2/-9 through activation of the ERK signaling pathway in HUVEC. Because of its therapeutic potential in diseases caused by or associated with reduced blood flow, further investigations are warranted to investigate the physiological effect and pro-angiogenesis mechanism of irisin in rodents and humans.

## Supporting Information

S1 Checklist NC3Rs ARRIVE Guidelines Checklist for zebrafish experiments. (PDF)

S1 Fig Effect of irisin on the phosphorylation of members of the MAPK family and AKT signaling pathways in HUVEC. (A) After treatment with or without irisin (10 and 20 nM) at the indicated time points in HUVEC, phosphorylated and total ERK, p38 and AKT levels in cell lysates were analyzed by Western blot. (B) HUVEC were pretreated with the ERK inhibitor U0126 for 30 min followed by irisin treatment (20 nM) for an additional 24 h, then the phosphorylated ERK, total ERK and β-actin protein expressions were analyzed by Western blot. (C) Protein bands were quantitated by densitometric analysis. Data are presented as the mean± SE of triplicates. ** P< 0.01, determined by unpaired two-tailed Student’s t-test.
(TIF)
