# Supplementary figures and images for: Irisin Induces Angiogenesis in Human Umbilical Vein Endothelial Cells In Vitro and in Zebrafish Embryos In Vivo via Activation of the ERK Signaling Pathway
Source: PLoS One. 2015 Aug 4;10(8):e0134662. doi: 10.1371/journal.pone.0134662 (PMC4524626; doi:10.1371/journal.pone.0134662)

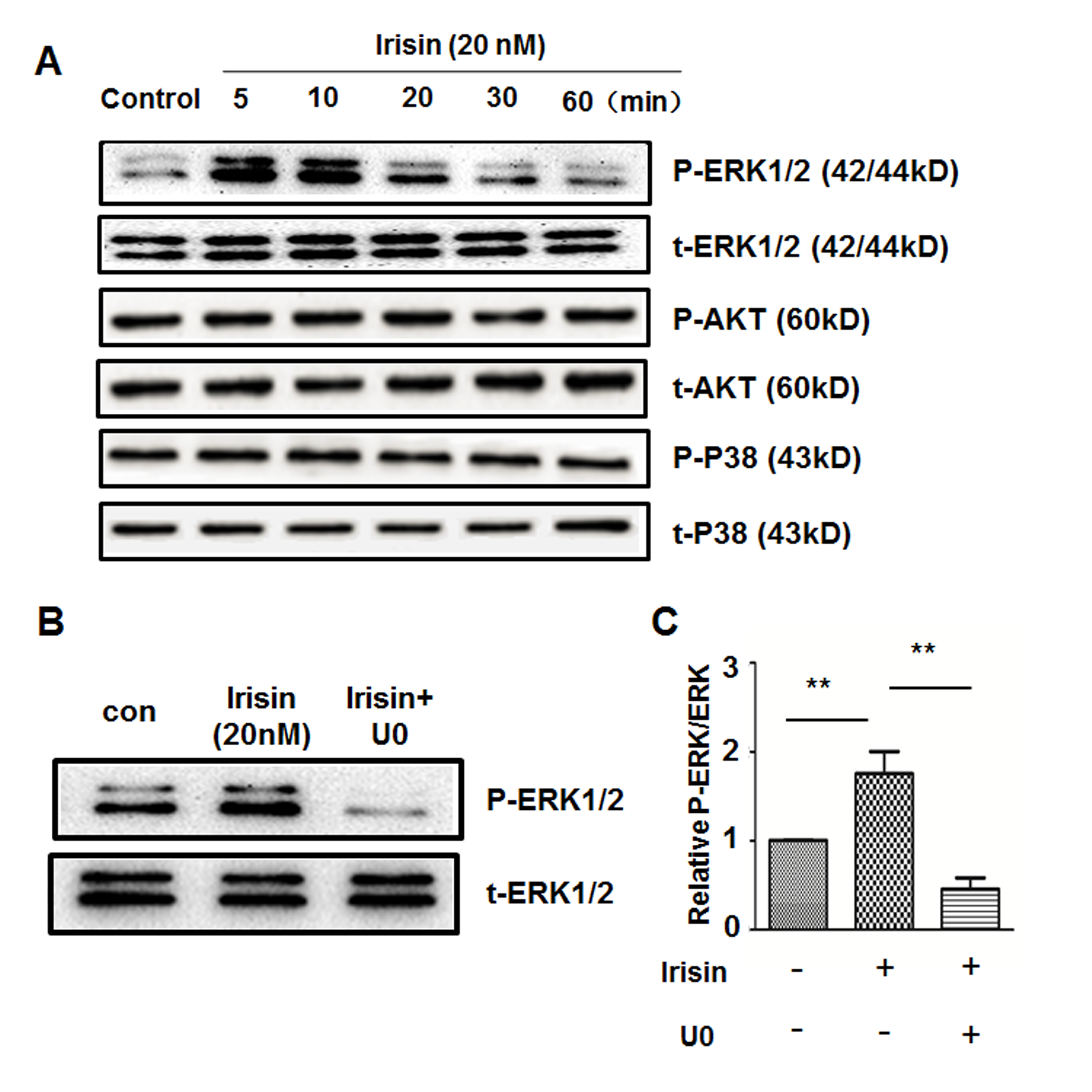

Supplement: S1 Fig — (A) After treatment with or without irisin (10 and 20 nM) at the indicated time points in HUVEC, phosphorylated and total ERK, p38 and AKT levels in cell lysates were analyzed by Western blot. (B) HUVEC were pretreated with the ERK inhibitor U0126 for 30 min followed by irisin treatment (20 nM) for an additional 24 h, then the phosphorylated ERK, total ERK and β-actin protein expressions were analyzed by Western blot. (C) Protein bands were quantitated by densitometric analysis. Data are presented as the mean± SE of triplicates. ** P< 0.01, determined by unpaired two-tailed Student’s t-test. (TIF) [file pone.0134662.s002.tif]
